# Performance Evaluation of an Autonomously Driven Agricultural Vehicle in an Orchard Environment

**DOI:** 10.3390/s22010114

**Published:** 2021-12-24

**Authors:** Joong-hee Han, Chi-ho Park, Young Yoon Jang, Ja Duck Gu, Chan Young Kim

**Affiliations:** 1 Division of Electronics & Information System, DGIST (Daegu Gyeongbuk Institute of Science and Technology), Daegu 42988, Korea; jhhan@dgist.ac.kr; 2 Sungboo IND Ltd., Chilgok 39909, Korea; chang1y@hanmail.net; 3 H&I (Human & Information), Uiwang 16009, Korea; 09jj@hni-gl.com (J.D.G.); 93cy@hni-gl.com (C.Y.K.)

**Keywords:** autonomous driving, agricultural vehicle, sensor fusion, GNSS, motion sensor

## Abstract

To address the problems of inefficient agricultural production and labor shortages, there has been active research to develop autonomously driven agricultural machines, using advanced sensors and ICT technology. Autonomously driven speed sprayers can also reduce accidents such as the pesticide poisoning of farmers, and vehicle overturn that frequently occur during spraying work in orchards. To develop a commercial, autonomously driven speed sprayer, we developed a prototype of an autonomously driven agricultural vehicle, and conducted performance evaluations in an orchard environment. A prototype of the agricultural vehicle was created using a rubber-tracked vehicle equipped with two AC motors. A prototype of the autonomous driving hardware consisted of a GNSS module, a motion sensor, an embedded board, and an LTE module, and it was made for less than $1000. Additional software, including a sensor fusion algorithm for positioning and a path-tracking algorithm for autonomous driving, were implemented. Then, the performance of the autonomous driving agricultural vehicle was evaluated based on two trajectories in an apple farm. The results of the field test determined the RMS, and the maximums of the path-following errors were 0.10 m, 0.34 m, respectively.

## 1. Introduction

According to a report from the Population Division of the United Nations Department of Economic and Social Affairs in 2019, the world’s population is projected to increase to 9.7 billion in 2050 [1], further increasing food demand in the future. At the same time, the agriculture sector currently faces problems because the population of farmers is aging and declining [2], and predictions indicate that it will be difficult to solve the shortage of food resulting from population growth. To address the global food shortage and problems in the agricultural sector, advanced agricultural countries are investigating autonomously driven agricultural vehicles, to replace the essential technology and labor required for agriculture with information and communication technologies (ICT), and intelligent sensor technology.

To operate autonomous driving, the vehicle must be equipped with sensors that provide either the navigation information or the surrounding environment information. The most popular sensors used in the operation of autonomous driving are GNSS, an inertial navigation system (INS), vision sensors, and laser scanners. The typical autonomous driving uses GNSS that can provide the position and velocity of a vehicle with an accuracy that is appropriate for the operation of autonomous driving. In the autonomous driving method using GNSS, the vehicle is driven along a predefined path by calculating both the steering angle and the wheel speed, based on waypoints using the vehicle’s current position and heading. However, since the drawback of GNSS is that its accuracy depends on the GNSS signal reception environment, autonomous driving using GNSS has the limitation that it is difficult to operate autonomous driving in all outdoor environments. To overcome the drawbacks of GNSS, it is combined with a dead-reckoning sensor, such as INS or motion sensors, which are not affected by the GNSS signal reception environment. The fusion of GNSS and a dead-reckoning sensor enables a stabler autonomous driving ability in an outdoor environment than when using only GNSS; however, the sensor’s disadvantage is that it cannot detect and avoid obstacles, because it cannot recognize external environment information. Since vision sensors can determine the vehicle’s dynamic state and the surrounding information, they are used in autonomous driving algorithms such as localization, map construction, path following, and obstacle avoidance. However, there are disadvantages in that image quality varies due to weather and illumination, and high computing power is required to process images in real-time. Unlike the method using a camera, the method using a laser scanner is less affected by weather and illuminance, but has disadvantages in that it has a high sensor price, and a large amount of data processing. Table 1 summarized the pros and cons of sensor types and application methods for autonomous driving.

Major agricultural machinery manufacturers have developed an autonomous driving system for an agricultural vehicle by appropriately combining the above-mentioned sensors (Table 2). The Yammar tractor producer presented a self-driving robot tractor, called “Yanmar Robot Tractor”, in 2019 [3]. This tractor operates with precise automatic driving control using a global navigation satellite system (GNSS), and an inertial measurement unit. The John Deere Company presented an autonomous tractor, the 8320 model in 2017 [4]. This model has a system called “Auto Trac Controller”, which allows it to adapt to the non-brand tractor with a plug and play kit, and it can detect obstacles using a laser scanner. The New Holland designed the “NHDrive”, an autonomous tractor that can work with complete autonomy [5]. The Case IH mentioned the concept of an autonomous tractor, which could be operated remotely using a tablet [6]. However, the currently developed autonomous driving agricultural vehicles use expensive sensors, so their penetration rate is low. Therefore, in order to increase the penetration rate of autonomous driving agricultural vehicles, it is considered necessary to develop autonomous driving agricultural vehicles composed of low-cost sensors.

Most existing autonomous driving agricultural machinery have used expensive navigation sensors relative to the price of agricultural machinery, so price competitiveness for commercialization is low. Therefore, this study developed a prototype for an autonomous driving agricultural vehicle including autonomous driving hardware and software using a low-cost GNSS and motion sensor, to help commercialize an autonomously driven speed sprayer. Then, the performance of the developed autonomously driven agricultural vehicle was evaluated in an orchard environment. A description of related works for navigation sensor-based autonomous driving is presented in Section 2. The results of the development of the autonomous driving agricultural vehicle with its system are introduced in Section 3. The performance evaluation of autonomous driving in an orchard environment is given in Section 4. Conclusions and future works are given in Section 5.

## 2. Related Work

To develop an autonomously driven agricultural vehicle, it is necessary to equip the vehicle with sensors that can provide navigation information and solutions. The Global Navigation Satellite System (GNSS) is currently the most popular navigation sensor, and can provide precise navigational information such as position, speed, and heading in outdoor environments. It has been widely employed in the development of autonomously driven agricultural vehicles. Nørremark et al. [7] developed an autonomous GPS-based system for an autonomous tractor with a row–line following accuracy of less than 2.2 cm. Ünal and Topakci [8] developed a GPS-guided autonomous robot for precision farming; in field tests, the results indicated a linear target-point precision ranging from 10 to 12 cm, and a distributed target-point precision ranging from 15 to 17 cm. Alonso-Garcia et al. [9] evaluated the performance of autonomously guided agricultural tractors using low-cost GPS receivers, and the total guidance error was lower than 1.25 m in 75% of the desired trajectories. Han et al. [10] developed a single-frequency GNSS RTK-based autonomous driving system, and its performance while driving autonomously on a parking lot was evaluated to have centimeter-level accuracy in path-following.

However, GNSS cannot provide continuous and stable positioning information in all agricultural environments; it has a low output rate, and does not provide attitude information, so there are limits to its application in autonomous driving technique development. An inertial navigation system (INS) can provide continuous navigation solutions with a high sampling rate, by using the acceleration and angular rate measured by an inertial measurement unit (IMU) without external disturbance [11]. However, the accuracy of the INS navigation solutions will degrade rapidly over time, due to the accumulation of sensor errors. As an alternative, a combination of GNSS and INS positioning techniques have been used in the development of autonomous driving techniques, taking advantage of their complementary features. Xiang et al. [12] developed an automatically guided rice transplanter using GNSS and IMU. The results in [12] showed that lateral and heading errors were less than 10 cm and 5 degrees on straight paths, respectively. Li et al. [13] studied autonomous navigation and path-tracking control on field roads in hilly areas, using a GNSS and INS fusion. In [13], the maximum deviation in the horizontal direction was 12.2 cm, and the average deviation was 5.3 cm. Han et al. [14] utilized GNSS and motion sensors to autonomously drive a crawler-type agricultural vehicle, and its performance in autonomous driving was evaluated to be within a 10 cm-level of accuracy. The previous studies using navigation sensors are summarized in Table 3.

Recently, various GNSS satellites, such as the global positioning system (GPS), the global navigation satellite system (GLONASS), Beidou, Galileo, and Quasi-Zenith Satellite System (QZSS) have begun operation, improving the GNSS reception environment. In addition, with advances in positioning technology using multiple GNSS, the performance of low-cost GNSS modules has also been improved. With further advances in microelectromechanical system (MEMS) technology, a small and cheap MEMS-based motion sensor has been developed with navigation-grade performance. Therefore, as a follow-up to prior research [14], we developed an autonomously driven agricultural vehicle, with a system based on low-cost navigation sensors, including a low-cost embedded board. Its performance was then evaluated as follows.

## 3. Materials and Methods

### 3.1. Autonomous Driving System Architecture

**Figure 1:**
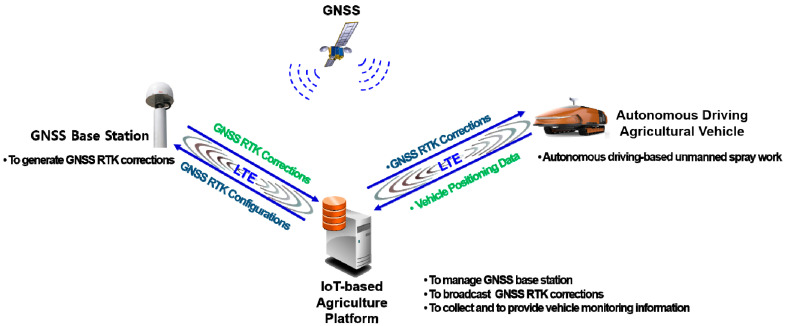
Autonomous driving system architecture.

The autonomous driving system architecture consists of a GNSS base station, an Internet of Things (IoT)-based agriculture platform, and the autonomous driving agricultural vehicle, as shown in Figure 1. The GNSS base station comprised a multi-band GNSS antenna, a GNSS module, and an LTE module. The role of the GNSS base station was to provide error data for GNSS raw observations. These were calculated by comparison with a known-location GNSS base station to operate the GNSS RTK. The IoT-based agriculture platform was used to manage the GNSS base station, to broadcast the correction data of the GNSS RTK, and to collect and provide information monitoring the locations of the autonomously driven agricultural vehicle. The autonomously driven agricultural vehicle operates an autonomous driving-based unmanned sprayer, using a predefined work path and real-time locations. Long-term evolution (LTE) with system components was used for data communication.

### 3.2. Autonomous Driving Agricultural Vehicle

**Figure 2:**
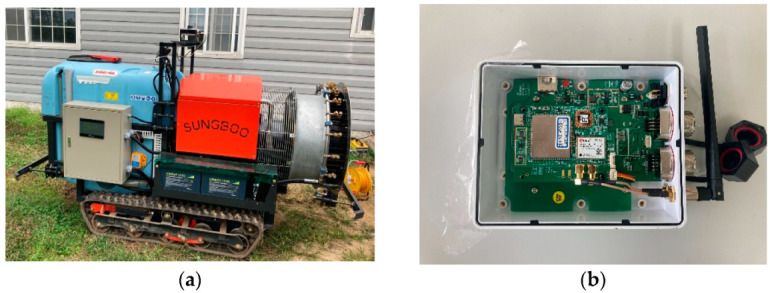
Autonomous driving agricultural vehicle (**a**) and autonomous driving hardware (**b**).

The autonomous driving agricultural vehicle used in this study was a prototype of a speed sprayer, and included an autonomous driving technique supplied by the Sungboo Industry Company, as shown in Figure 2a. It was a rubber-tracked vehicle, equipped with two 2.0 kW DC48V AC motors. The external dimensions were 2183 mm (length) × 1300 (width). Front and rear bumper sensors were installed to allow an emergency stop in the event of a collision during autonomous driving. The chemical liquid container used a 500 L tank, and it was equipped with a gasoline engine for spraying the chemical liquid. In addition, it included an alternator that could charge the electric battery of the driving system while spraying.

The vehicle was equipped with a prototype of autonomous driving hardware and a GNSS antenna. The autonomous driving hardware prototype (Figure 2b) consisted of a GNSS module, a motion sensor module, an embedded board, and an LTE module. The GNSS module used a u-blox ZED-F9P [15], and a low-cost multi-band GNSS module under $200 to provide stable and precise GNSS RTK positioning information in an orchard environment. The motion sensor module was Xsens’s MTi-1 [16], which contained a 3-axis accelerometer, a 3-axis gyroscope, and a 3-axis magnetometer. The price of the MTi-1 was less than $200, and it was among the low-cost modules available for precise navigation. An embedded board and the raspberry pi 4 model were used to operate the autonomous driving control software. The GNSS antenna used Hi-Target’s AH-4236 [17] which supported GPS, GLONASS, Beidou, Galileo, and L-Band signal reception. The price of the AH-4236 was about $100.

### 3.3. Autonomous Driving Control Software

**Figure 3:**
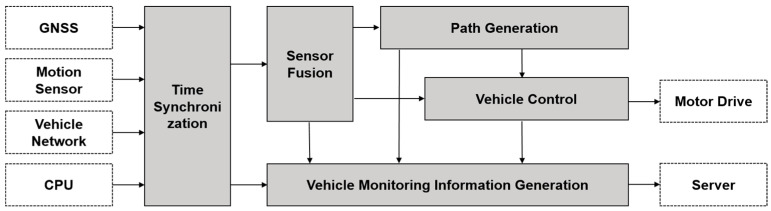
Functional architecture of autonomous driving control software.

The developed autonomous driving control software for operating the autonomously driven agriculture vehicle consisted of five independent modules: (1) a time synchronization; (2) sensor fusion; (3) path generation; (4) vehicle control; (5) vehicle monitoring information generation (Figure 3). The time synchronization was used to synchronize both the CPU time of the motion sensor data and the vehicle status information acquired through the vehicle network with the GPS time. The detailed process for time synchronization is explained in Section 3.4. The sensor fusion system calculated navigational information by using the GNSS and motion sensor data to operate path generation and vehicle control. The details of the sensor fusion are described in Section 3.5.

The path generation defined waypoints for autonomous driving based on navigational information acquired by the user, by manually driving the vehicle. The vehicle control calculated the motor control parameters including the left and the right track RPM, and sent parameters by RS422 to the motor drive for autonomous driving along the desired path, based on the waypoints and navigational information. The process of path generation and vehicle control is described in Section 3.6 and Section 3.7, respectively. The vehicle monitoring information is used to generate information to monitor the operation of the agricultural vehicle. The outputs of this function are the navigation data, the operational status of the agricultural vehicle, and the waypoints. These outputs are transmitted by LTE to the server to monitor the agricultural vehicle.

### 3.4. Time Synchronization Algorithm

Time synchronization of the sensor data was essential for optimal sensor fusion and reliable monitoring. Since the motion sensor data and the vehicle status data acquired through the vehicle network used in this study were not time-tagged data, an algorithm for synchronizing with GPS time was developed using CPU time, and the GPS time in the GNSS data. The time synchronization algorithm process worked as follows. The first step stored the GPS time of the GNSS data and the CPU time of the embedded OS at the time the GNSS data was received, for 10 s. Then, the difference between the GPS time and CPU time was calculated using the data stored in the first step. At this step, there may have been an outlier in the GPS time or the CPU time, so if the difference between the GPS time and the CPU time was three sigma or more, it was assumed to be an outlier and deleted. The offset between the GPS time and the CPU time was determined by the average of the difference between the GPS time and the CPU time. Finally, the GPS time in the motion sensor data and the vehicle status data were defined using the following equation:

(1) $$T_{GPS}=T_{CPU}-O_{GNSS-CPU},$$

where *T_GPS_* is the GPS time of the motion sensor data or the vehicle status data, *T_CPU_* is the CPU time at the time of data acquisition, and *O_GNSS-CPU_* is the offset between the GPS time and the CPU time.

### 3.5. Sensor Fusion Algorithm

For stable autonomous driving and unmanned work in an orchard environment, it is necessary to continuously provide accurate navigational information to the agricultural vehicle in real-time. Although GNSS-RTK positioning provides a precise location within several centimeters, it has a disadvantage in that it can provide inaccurate positioning information due to poor GNSS signals, or signal blocking by fruit trees and leaves in an orchard environment. Dead reckoning using an Inertial Measurement Unit (IMU), and a motion sensor including a magnetometer, can provide a navigation solution with a continuously high observation rate that will not be affected by the surrounding environment. However, the error of the navigation solution will sharply increase due to the accumulation of sensor errors over time. Table 4 summarizes the pros and cons of the GNSS-based positioning, and the motion sensor-based dead reckoning. Combining the GNSS and motion sensor can improve the accuracy and reliability of navigational solutions because of the complementary natures of the motion sensor-based dead reckoning and GNSS-based positioning.

Therefore, in this study, to provide stable and continuous navigational information in an orchard environment, a real-time multi-sensor fusion-positioning algorithm that combined the motion sensor and GNSS, was developed.

**Figure 4:**
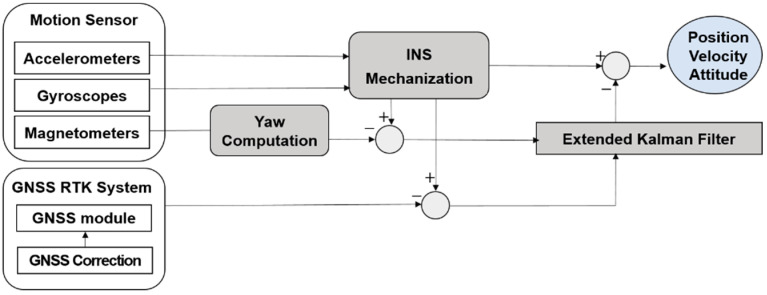
Block diagram of GNSS and motion sensor fusion.

A block diagram of the proposed sensor fusion algorithm is shown in Figure 4. The proposed algorithm was implemented with loosely coupled integration though an extended Kalman filter (EKF). The process of the proposed algorithm was as follows. First, the navigation information (position, velocity, and attitude) was calculated through the inertial navigation system (INS) using the accelerations and angular velocities provided by the motion sensor at intervals of 0.01 s. In addition, the magnetometers’ measurements were simultaneously provided with the accelerations and angular velocities, yaw was calculated using position, attitude, and magnetic declination, and then the magnetometer-based yaw update was carried out. If the GNSS provided the position and the velocity, a GNSS update was conducted.

### 3.6. Path-Generation Algorithm

In general, an autonomous driving path can be generated by defining waypoints, which is a route for an agricultural vehicle to autonomously drive on a map. However, there is a limit to the map-based autonomous driving path-generation method, since most areas where agricultural vehicles are driven do not have precise maps, and the cost of surveying for precise map construction is very high. In this study, we developed a path-generation algorithm to generate waypoints using navigational information obtained by manually driving a route, so that the vehicle could autonomously drive in an area without a map.

**Figure 5:**
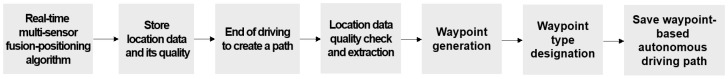
Process of the path generation algorithm.

The process of the proposed path generation algorithm was as follows (Figure 5). First, when a user manually drove an autonomous driving route, location data and its quality information obtained from the real-time multi-sensor fusion-positioning algorithm were stored. When the user completed driving for path generation, the location data with good quality was extracted. In this study, the location data were regarded as good quality when the age of the GNSS measurement update with the resolved ambiguity did not exceed 2 s and the precision of location was lower than 0.5 m.

**Figure 6:**
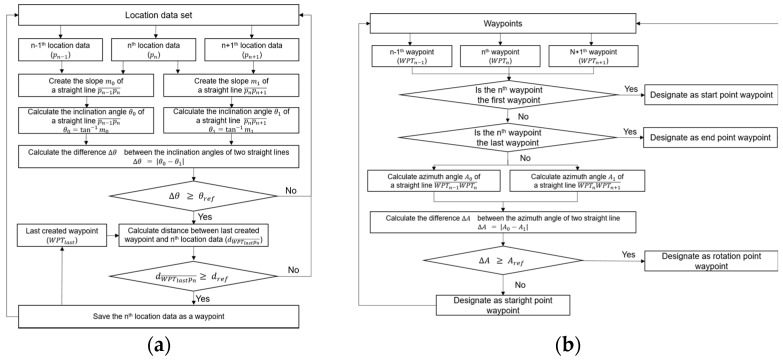
Flow chart of waypoint generation (**a**) and waypoint designation type (**b**).

Next, a waypoint was created using the location data with good quality, and two parameters of the distance (d_ref_) between adjacent points and the angle (θ_ref_) between successive lines were calculated using the adjacent points (Figure 6a). Then, based on the reference angle for defining the rotation point, the type of waypoint was designated by dividing the straight point and the rotation point (Figure 6b). Finally, waypoint data including waypoint number, geodetic coordinates (latitude, longitude, and ellipsoidal height), waypoint type, azimuth of a straight line created by adjacent waypoints, and angles between straight lines created by adjacent waypoints were output.

### 3.7. Vehicle Control Algorithm

**Figure 7:**
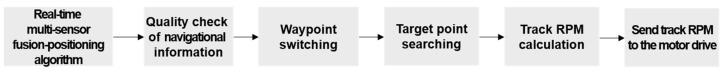
Process of the vehicle control algorithm.

The vehicle-control algorithm was used to calculate the left and the right track RPM, using waypoints and navigational information, and then to send the left and the right track RPM to the motor drive for autonomous driving along the desired path. The vehicle-control algorithm consisted of four main functions: a quality check of navigational information; a waypoint switching; target point searching; and track RPM calculation. The process of the proposed vehicle control algorithm is shown in Figure 7.

A quality check of the navigational information step was used to determine whether the agricultural vehicle moved or stopped, using the quality information of the navigational information calculated by the real-time multi-sensor fusion-positioning algorithm. In this study, when the age of the GNSS measurement update with the resolved ambiguity did not exceed 2 s, and the precision of location was lower than 0.5 m, the quality of navigational information was regarded as good. If the navigation solution quality was good, the vehicle-control algorithm proceeded to the next step; otherwise, a stop command was sent to the motor drive, until the navigation solution quality was good.

**Figure 8:**
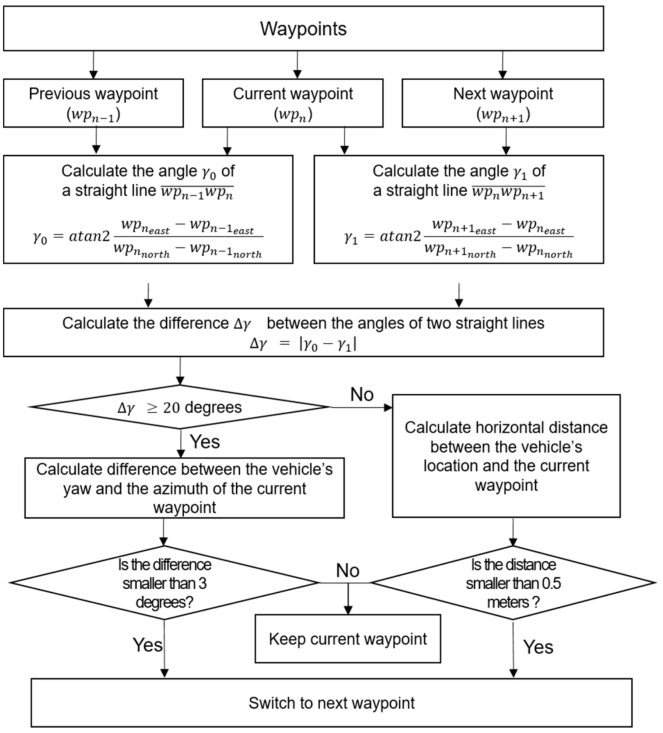
Waypoint switching process.

In the waypoint switching step, it was decided whether to continue using the current waypoint or to switch to the next waypoint. While autonomously driving, the agricultural machinery moved between successive waypoints and, to improve stability, it was necessary to appropriately switch waypoints based on the current location. In general, if the distance between the vehicle location and the currently selected waypoint was within a certain distance, the waypoint was switched. However, since this study used a crawler-type agricultural vehicle, it additionally considered whether the current waypoint was the point of in-situ rotation or not. The waypoint-switching process is given in Figure 8.

In the target-point searching step, the enclosed baseline of the sight guidance method [18] was applied. A target point was a point intersection between a circle with radius R enclosing the current vehicle’s location, and a straight line created from the previous waypoint to the current waypoint. To calculate the coordinates of a target point, a detailed formula was written in [18]. In the track RPM calculation step, the RPM of the left and right tracks needed to reach the target point at the next epoch, calculated by dividing it into two cases of rotation and movement in place. In the first case, the vehicle rotated in-situ when the currently selected waypoint was a point of in-situ rotation, and the distance between the current vehicle’s location and the current waypoint’s location was smaller than 0.4 m. In this case, the magnitude of the left and right track RPM was set as 100, and the signs of the left and right track speed were set differently, depending on the rotation direction. In the other case, the left and right track RPM were calculated using a scale that converted the track speed into RPM, after calculating the speed and steering angle needed to reach the target point using the target location, the vehicle’s current location, and its yaw. The detailed method for the track RPM calculation is explained in [14].

## 4. Performance Evaluation of Autonomous Driving in an Orchard Environment

### 4.1. Test Description

**Figure 9:**
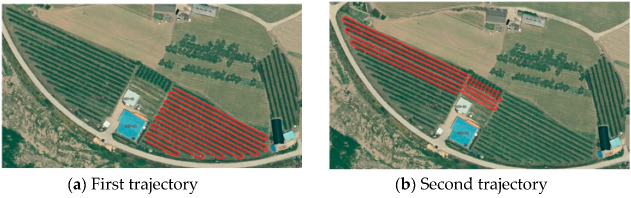
Two trajectories (red lines) used to evaluate autonomous driving performance.

To evaluate the autonomous driving performance, field tests were performed with two different trajectories. The experiment for autonomous driving was conducted on an apple farm (YoungCheon, Korea). Figure 9 shows the two trajectories, shown as a red line overlaid on the aerial photos. The two trajectories included various driving circumstances encountered during typical driving on an apple farm. Apple trees were arranged on the left and right sides of the straight path in the trajectories. The trajectories were 3.5 m wide, and rotation sections had large curvatures.

The configurations of the module and the software used for the test were as follows. The configuration of the GNSS module was set to receive GPS, GLONASS, Galileo, Beiduo, and QZSS. The GNSS positioning method was set to the single baseline GNSS RTK mode and the output rate of the GNSS data was set to 5 Hz. The output rate of the motion sensor module was set to 100 Hz, which was the maximum output rate of the motion module used. The control interval of the vehicle-control algorithm was set to 0.01 s, equal to the output rate of the motion sensor module and the real-time multi-sensor fusion-positioning algorithm. The maximum speed was set at 6 km/h, considering the driving speed of the speed sprayer work. The acceptance radius for switching waypoints and the radius for searching a target point were set to 0.4 m and 2 m, respectively. The data used to generate the waypoints of the two trajectories were acquired by manually driving the autonomous driving agricultural vehicle using a wireless remote controller. To generate waypoints with the path-generation algorithm, the two parameters of the minimum distance between adjacent points and the angle between successive lines were set to 0.5 m and 3 degrees, respectively. It was set to finish the operation of autonomous driving when the distance between the last waypoint and the vehicle’s location was within 0.3 m.

The performance of autonomous driving was evaluated by calculating the path-following error and the distance between the waypoint and the vehicle’s location, respectively. The path-following error was calculated as the shortest distance between a vehicle’s location and a straight line between two consecutive waypoints at every epoch. The distance between the waypoint and vehicle’s location was calculated as the shortest distance between a waypoint and the vehicle’s location at every waypoint.

### 4.2. Performance Evaluation in the First Trajectory

**Figure 10:**
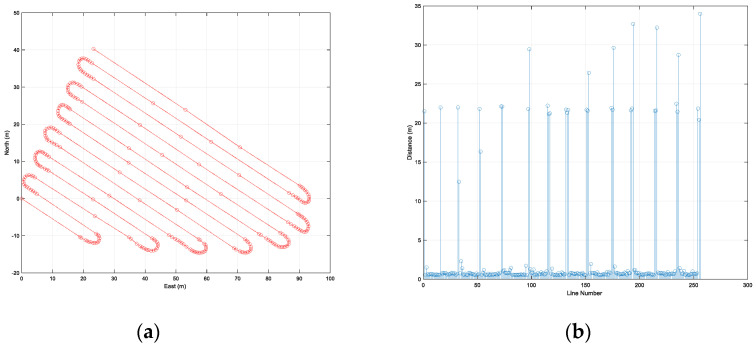
Waypoints of the first trajectory (**a**) and the distance between adjacent waypoints (**b**).

Figure 10 shows the waypoints of the first trajectory expressed using a north, east, and down (NED) relative coordinate system with the origin at the first waypoint, and the distance between adjacent waypoints. The first trajectory included 14 straight-line sections and 13 curved sections. The first trajectory contained 257 waypoints, and the length of the first trajectory was 946 m. The range of distance between adjacent waypoints was from 0.5 m to 34 m.

**Figure 11:**
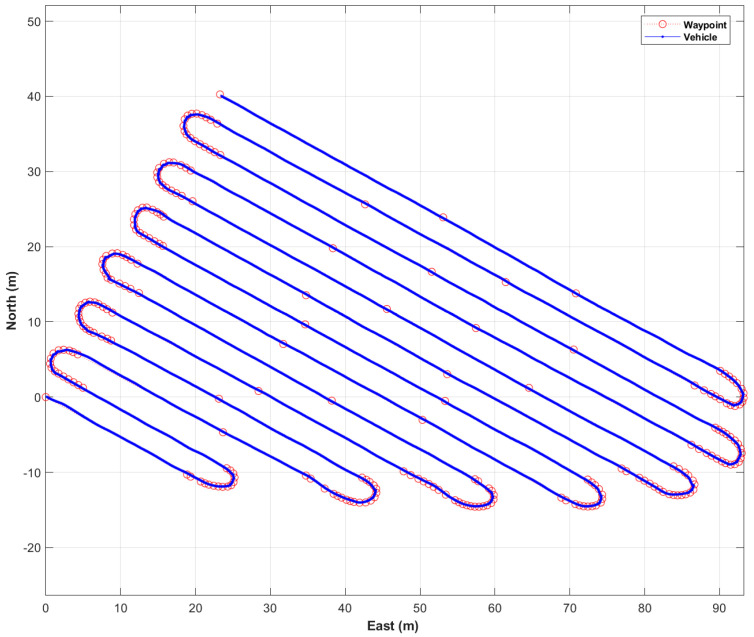
Autonomous driving results in the first trajectory: vehicle locations traveled by autonomous driving (blue points) and waypoints (red circles).

**Figure 12:**
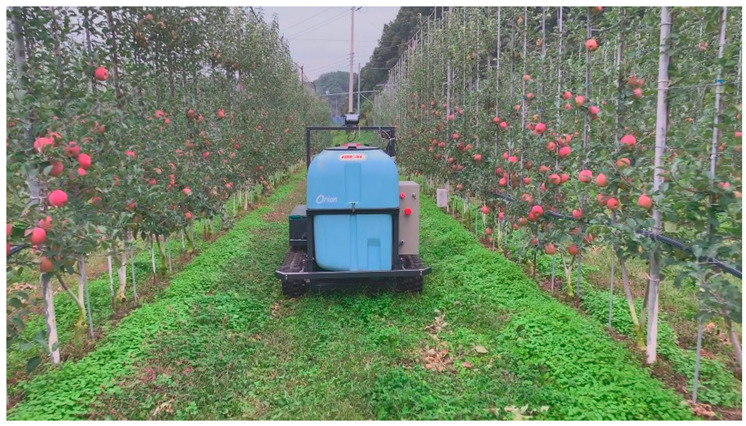
The autonomous driving agricultural vehicle operating in the first trajectory.

Figure 11 shows the vehicle locations traveled by autonomous driving (blue points) and waypoints (red circles). Figure 12 is a picture of a vehicle that is driving autonomously in a straight section. The autonomous driving operation time for the first trajectory was 25 min 45.47 s. The maximum and the average speed of the vehicle during autonomous driving was about 6 km/h and about 3.5 km/h, respectively. In addition, the distribution of vehicle attitude was −2 to 6 degrees for roll and −2 to 7 degrees for pitch.

**Figure 13:**
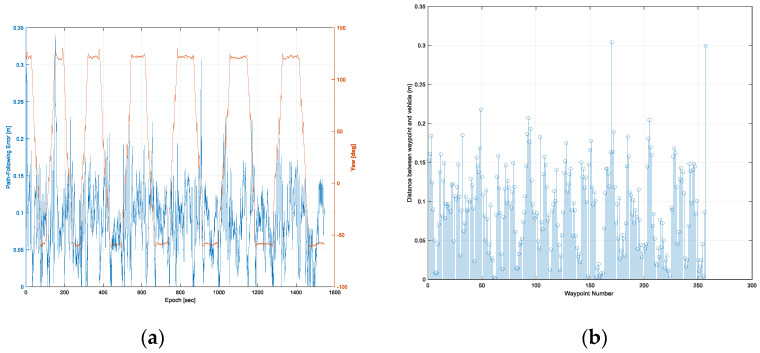
Results of autonomous driving performance evaluation in the first trajectory: the path-following error at every epoch (**a**), and the distance between a waypoint and a vehicle’s location at every waypoint (**b**).

The path-following error and the vehicle’s yaw at every epoch are shown in Figure 13a. In the first trajectory, the maximum error of the path-following was calculated to be 0.34 m, and the RMS of the path-following error was 0.10 m. It was found that the path-following error of the rotation sections was higher than that of the straight sections. This was caused by track slip, due to both vehicle dynamic force and the ingress of soil inside the track during in-situ rotation.

**Figure 14:**
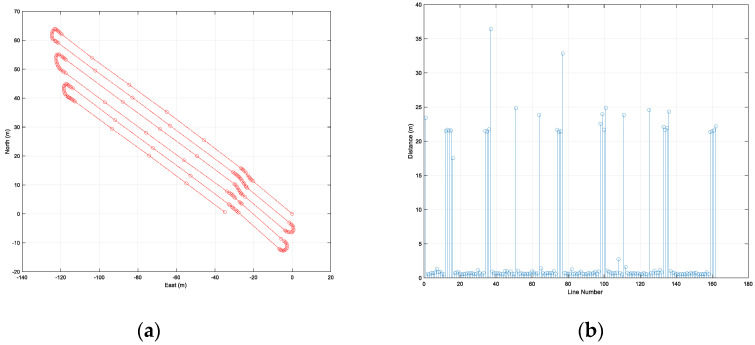
Waypoints of the second trajectory (**a**), and the distance between adjacent waypoints (**b**).

Figure 14b shows the distance between a waypoint and a vehicle’s location at every waypoint. The range of the distance between a waypoint and a vehicle’s location was 0.002 to 0.30 m. The average and RMS of the distance between a waypoint and a vehicle’s location were 0.09 and 0.11 m, respectively. Two points with a distance of about 0.3 m were a waypoint of the rotation section and the last waypoint, respectively.

### 4.3. Performance Evaluation in the Second Trajectory

The second trajectory included six straight-line sections and five curved sections containing 163 waypoints (Figure 14). The length of the first trajectory was 788 m, the range of distance between adjacent waypoints was from 0.5 m to 36 m. Unlike the first path, the second path had a curved line in the straight section.

**Figure 15:**
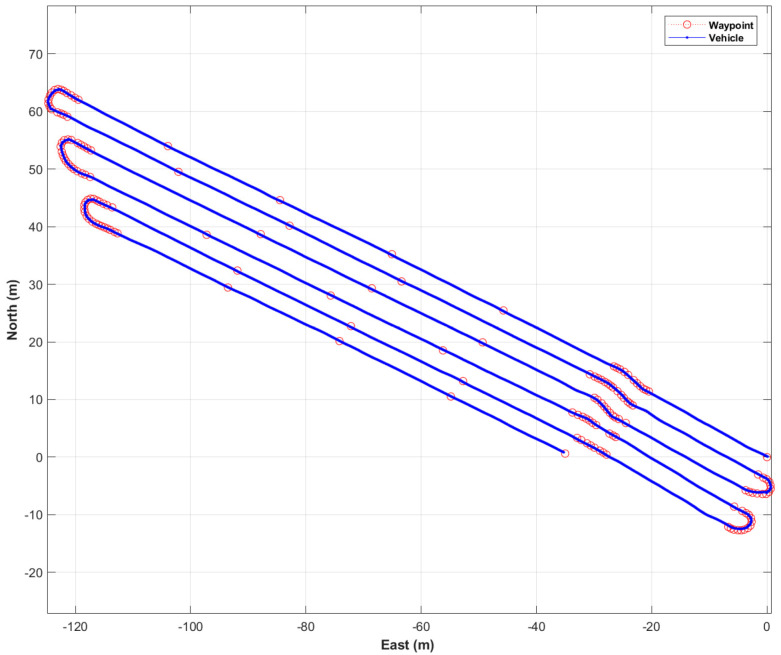
Autonomous driving results in the second trajectory: vehicle locations traveled by autonomous driving (blue points) and waypoints (red circles).

**Figure 16:**
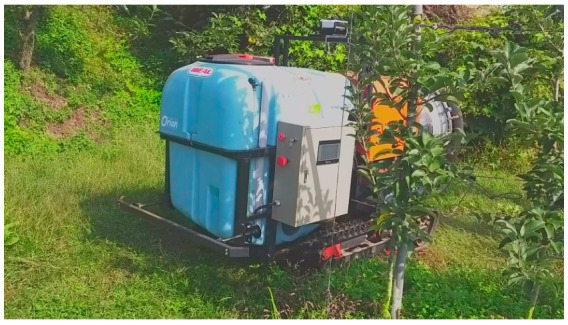
The autonomous driving agricultural vehicle operating in the second trajectory.

Figure 15 and Figure 16 show the vehicle location with waypoints for the second trajectory, and a picture of a vehicle autonomously driving in a rotation section. The autonomous driving operation time for the second trajectory was 17 min 54.3 s. The maximum and the average speed of the vehicle during autonomous driving was 6 km/h and 2.7 km/h, respectively. As the distribution of vehicle attitude was −4 to 4 degrees for roll and −5 to 8 degrees for pitch, the second trajectory had a more undulating path than the first trajectory.

**Figure 17:**
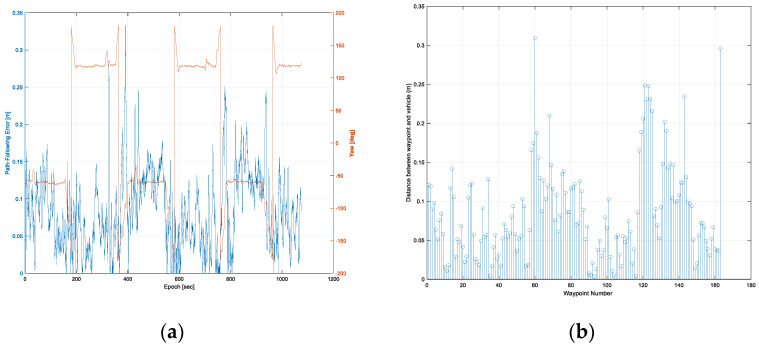
Results of autonomous driving performance evaluation in the second trajectory: the path-following error at every epoch (**a**), and the distance between a waypoint and a vehicle’s location at every waypoint (**b**).

Figure 17a,b show the path-following error with the vehicle’s yaw and the distance between a waypoint and the vehicle’s location, respectively. The maximum error and RMS of the path-following were 0.33 and 0.10 m, respectively. In the second trajectory, similar to the results of the first trajectory, a larger error occurred in the rotation section than in the straight section. The range of distance between a waypoint and a vehicle’s location was 0.003 to 0.31 m. The average and RMS of the distance between a waypoint and a vehicle’s location were 0.09 and 0.10 m, respectively. Two points with a distance of about 0.3 m were a waypoint of the rotation section and the last waypoint, respectively. As can be seen from the above results, the autonomous driving performance in the first trajectory and the second trajectory were similar.

## 5. Conclusions and Future Works

This study presents the results of the development of autonomous driving hardware and software, for a commercial speed sprayer autonomously driven agricultural vehicle. A prototype of an agricultural vehicle was made using a rubber-tracked vehicle equipped with two 2.0 kW AC 48 V motors. A prototype of the autonomous driving hardware was made including a GNSS module, a motion sensor, an embedded board, and an LTE module. Its manufacturing cost was less than $1000. The autonomous driving software consisted of a time-synchronization algorithm, a sensor fusion algorithm, path-generation algorithm, vehicle-control algorithm, and a vehicle-monitoring information-generation algorithm. To evaluate the performance of the autonomously driven agricultural vehicle, we conducted field tests using two waypoint-based trajectories on an apple farm. The results showed that the RMS and the maximum of the path-following error, with respect to two trajectories, were 0.10 m and 0.34 m, respectively. The average and RMS of the distance between a waypoint and a vehicle’s location were 0.09 and 0.11 m, respectively. In addition, the results of autonomous driving performance using the two trajectories were almost the same, indicating stable autonomous driving. Therefore, based on these results, it can be concluded that it is possible to develop a commercial autonomously driven agricultural vehicle using low-cost navigation sensors.

To commercialize the autonomous driving speed sprayer, further tasks will be required, as follows. Since a spray test, which is the main task of the speed sprayer, was not carried out, we will develop an autonomous driving-based spray technique to analyze its efficiency, compared to the existing manual method. In addition, since the performance of autonomous driving depends on orchard environments, we will conduct an additional analysis based on a wide variety of farm conditions, and carry out steps to further stabilize the operation of the machines and algorithms. Furthermore, based on the results of various experiments, to prevent the vehicle from overturning when the terrain is irregular, we will add a method to send a stop command to the vehicle when the roll and pitch values above a certain value are calculated. Since the developed agricultural vehicle is only equipped with a bumper sensor to stop after a collision, we are considering equipping ultrasonic sensors to prevent collisions at the lowest cost; we plan to equip the vehicle and conduct an according experiment in the future. In addition, to improve the safety and the performance of autonomous driving operations, we will be conducting additional research on whether to apply camera or LiDAR-based SLAM technology. Moreover, if the LTE network coverage problem occurs in future experiments, we will consider applying alternative communication technology such as Zigbee or LoRa. Finally, we will plan to develop a user-friendly app and an IoT agriculture platform to help farmers easily use the autonomously driven speed sprayer.

## Figures and Tables

**Table 1:** The pros and cons of sensor types and application methods for autonomous driving.

Type	Pros	Cons	Application Methods
GNSS	● Provides accurate position and velocity ● Low price compared with other sensors	● Accuracy depends on the GNSS signal reception environment ● Unable to perceive surrounding situations	● Localization ● Path generation ● Path following
Fusion of GNSS and dead-reckoning sensors	● Provides continuously accurate navigation solutions (position, velocity, attitude)	● Accuracy depends on the performance of dead-reckoning sensors ● Unable to perceive surrounding situations	● Localization ● Path generation ● Path following
Visionsensor	● Provides vehicle’s dynamic states and surround information ● Low price compared with Laser scanner	● Information quality varies due to weather and illumination ● Needs high computing power	● Localization ● Map construction ● Path following ● Obstacle avoidance
Laserscanner	● Provides vehicle’s dynamic states and surround information ● Less affected by weather and illuminance	● Needs high computing power ● Higher price compared with agricultural machinery	● Localization ● Map construction ● Path following ● Obstacle avoidance

**Table 2:** Summary of autonomous driving agricultural vehicles.

Manufactures	Vehicle Type	Sensors	Level
Yammar [3]	Tractor	GNSS, IMU, laser sensor, ultrasonic sensor	Autonomous driving commercialization
John Deere [4]	Tractor	GNSS, laser scanner	Autonomous driving commercialization
New Holland [5]	Tractor	GNSS, LiDAR, camera	Autonomous driving commercialization
Case IH [6]	Tractor	GNSS, radar, camera	Concept of autonomous driving
This paper	Speed Sprayer	A low-cost GNSS and motion sensor	Prototype of autonomous driving

**Table 3:** Summary of previous studies using navigation sensors.

Reference	Vehicle Type	Sensors	Performance
Nørremark et al. [7]	Tractor	Two GPS	Less than 2 cm
Ünal and Topakci [8]	Robot for precision faming	GPS	10 to 12 cm
Alonso-Garcia et al. [9]	Tractor	GPS	Less than 1.25 m
Han et al. [10]	Speed sprayer	Single-frequency GNSS	Centimeters
Xiang et al. [12]	Rice trans planter	GNSS and IMU	Less than 10 cm
Li et al. [13]	Small vehicle	GNSS and INS	5.3 cm
Han et al. [14]	Speed sprayer	GNSS and motion sensor	Centimeters

**Table 4:** The pros and cons of GNSS-based positioning and motion sensor-based dead reckoning.

Type	Pros	Cons
GNSS-based positioning	● The accuracy of positioning information does not change with time ● No initialization is required when positioning is conducted	● The accuracy of positioning information varies depending on the surrounding environment ● The data rate is low
Motion sensor-based dead reckoning	● The accuracy of positioning information is maintained without external signals for a short period of time ● The data rate is very high	● The error of positioning information increases as time passes, due to sensor errors and initial errors ● Initialization is required when positioning is conducted

## Data Availability

Not applicable.
